# FOXQ1-mediated SIRT1 upregulation enhances stemness and radio-resistance of colorectal cancer cells and restores intestinal microbiota function by promoting β-catenin nuclear translocation

**DOI:** 10.1186/s13046-021-02239-4

**Published:** 2022-02-19

**Authors:** Mei Yang, Qian Liu, Maolin Dai, Renqun Peng, Xinghui Li, Wei Zuo, Juhua Gou, Feixue Zhou, Shuangjiang Yu, Hao Liu, Min Huang

**Affiliations:** 1 Department of Digestion, Rongchang District People’s Hospital of Chongqing, No. 3, Guangchang North Road, Changyuan Street, Chongqing, 402460 China; 2 The Hospital of Chongqing University of Posts and Telecommunications, Chongqing, 400065 China; 3 Department of Anesthesiology, Rongchang District People’s Hospital of Chongqing, Chongqing, 402460 China; 4 Department of Neurosurgery, The First Hospital Affiliated to Army Military Medical University (Southwest Hospital), Chongqing, 400038 China; 5 Department of Digestion, Affiliated Hospital of North Sichuan Medical College, No. 63, Wenhua Road, Shunqing District, Nanchong, 637000 Sichuan Province China

**Keywords:** Colorectal cancer, Intestinal bacteria, Stemness of cancer cell, Radiation resistance, Forkhead box Q1, SIRT1, Deacetylation, β-catenin, Nuclear translocation

## Abstract

**Background:**

Resistance of colorectal cancer (CRC) cells to radiotherapy considerably contributes to poor clinical outcomes of CRC patients. Microarray profiling in this study revealed the differentially expressed forkhead box Q1 (FOXQ1) in CRC, and thus we aimed to illustrate the role of FOXQ1 in CRC by modulating stemness and radio-resistance of CRC cells.

**Methods:**

CRC and adjacent normal tissues were collected from CRC patients, and the correlation between FOXQ1 expression and CRC prognosis was analyzed. Subsequently, we determined the expression of FOXQ1, sirtuin 1 (SIRT1) and β-catenin in CRC tissues and cell lines. The binding affinity between FOXQ1 and SIRT1 and that between SIRT1 and β-catenin were validated with luciferase reporter gene, Co-IP and ChIP assays. Following a metagenomics analysis of CRC intestinal microbiota, the effects of the FOXQ1/SIRT1/β-catenin axis on CRC stem cell phenotypes and radio-resistance was evaluated *in vitro* and *in vivo* through manipulation of gene expression. Besides, mouse feces were collected to examine changes in intestinal microbiota.

**Results:**

FOXQ1 was highly expressed in CRC tissues and cells and positively correlated with poor prognosis of CRC patients. FOXQ1 overexpression contributed to resistance of CRC cells to radiation. Knockdown of FOXQ1 inhibited the stemness of CRC cells and reversed their radio-resistance. FOXQ1 enhanced the transcriptional expression of SIRT1, and SIRT1 enhanced the expression and nuclear translocation of β-catenin. Knockdown of FOXQ1 repressed SIRT1 expression, thus reducing the stemness and radio-resistance of CRC cells. Moreover, FOXQ1 knockdown suppressed CRC xenograft formation in xenograft-bearing nude mice through inhibiting SIRT1 and β-catenin to reduce the content of pathological bacteria that were up-regulated in CRC.

**Conclusion:**

FOXQ1-mediated SIRT1 upregulation augments expression and nuclear translocation of β-catenin and benefits CRC-related intestinal pathological bacterial, thereby enhancing the stemness and radio-resistance of CRC cells.

**Supplementary Information:**

The online version contains supplementary material available at 10.1186/s13046-021-02239-4.

## Background

Colorectal cancer (CRC) represents a common malignancy and one of the leading causes of cancer-related death worldwide [1]. In spite of progress made in regard of the prevention and treatment for CRC, many patients still experience tumor recurrence and metastasis with existing therapies, including radiotherapy and chemotherapy [2–4]. It has been demonstrated by genomic studies that CRC shows obvious heterogeneity, and that various epigenetic modifications regarding the heterogeneity of CRC stem cells occur in the pathogenesis of CRC [5]. Of note, stem cells are also responsible for the resistance to radiotherapy in CRC [6, 7]. Moreover, the functions and phenotypes of tumor stem cells are mainly determined by the tumor microenvironment [8]. Herein, understanding the mechanisms underlying the stemness and radio-resistance in CRC cells is imperative for the development of strategies targeting CRC stem cells, and thus to prevent CRC recurrence and enhance the prognosis in CRC patients.

Tissue microarrays allow molecular profiling of novel diagnostic/predictive markers in cancer specimens on the basis of quantitative genomic and proteomic analyses of tumor samples [9, 10]. In this study, we searched out two representative CRC-related microarrays from the GEO database, the GSE21510 [11] with abundant tissue samples (n = 148) and the relatively new GSE156355 published on August, 2020 [12]. Both of the two microarrays comprised samples from CRC patients and healthy controls, and preliminary differential analysis of them revealed Forkhead box Q1 (FOXQ1) and KRT23 to be obviously up-regulated genes in CRC. Intriguingly, the occurrence of FOXQ1 overexpression in CRC cell line HCT116 and HT29 has been documented [13]. Furthermore, the up-regulated expression of FOXQ1 has been correlated with tumor stem cell radio-resistance in pancreatic cancer [14]. Thus, we speculated that FOXQ1 may also has a role to play in CRC stem cell radio-resistance.

Moreover, FOXQ1 has been found to augment the transcriptional expression of sirtuin 1 (SIRT1) deacetylase [15]. The mammalian sirtuin family of proteins has been the focus of many studies due to their recognized regulatory functions in a variety of cellular processes, including cellular metabolism and aging [16]. More importantly, SIRT1 overexpression has been indicated in CRC [17]. In addition, a previous study has correlated SIRT1 with resistance to radiotherapy [18]. Further, SIRT1-mediated deacetylation triggers the nuclear translocation and activity of the transcription factor β-catenin [19]. Accumulating evidence has suggested that abnormal regulation of β-catenin could result in early events in carcinogenesis [20, 21]. Interestingly, β-catenin has been highlighted as a regulator of the transcription of FOXQ1 [22]. It has also been indicated that β-catenin may participate in the resistance of glioma cell to radiotherapy [23]. Moreover, a potential correlation between β-catenin-related pathways and intestinal microbiota has been previously suggested [24, 25]. Based on the aforementioned evidence, we proposed a hypothesis in the present study that the activation of FOXQ1/SIRT1/β-catenin regulatory circuit may promote CRC stem cell phenotypes to induce resistance to radiotherapy in an intestinal microbiota-related way.

## Methods

### Ethics statement

The study was conducted in accordance with the *Declaration of Helsinki* and approved by the Ethics Committee of Rongchang District People's Hospital of Chongqing. Detailed study aims as well as planned procedures were explained to all patients and their caregivers, who subsequently signed the informed consent. Animal experiments were approved by the Animal Care and Use Committee of Rongchang District People's Hospital of Chongqing and performed in accordance with *Guide for the Care and Use of Laboratory Animals* published by the National Institutes of Health.

### Bioinformatics analysis

CRC-related microarray datasets, GSE21510 (containing 19 CRC homogeneous tissue samples and 25 normal samples) and GSE156355 (containing 6 CRC tissue samples and 6 normal samples), were retrieved from the GEO database. Then, differentially expressed mRNAs in the two microarrays were identified using Limma package in R language with |logFC| > 5 and *p* < 0.01 as a screening threshold, the intersection of which were then obtained using Venn diagram and taken as candidate CRC-related genes. In addition, the expression of FOXQ1 in CRC and normal samples were validated using "boxplot" package in R language.

### Metagenomics analysis

A CRC-related metagenomic sequencing project, PRJNA397219, was retrieved from GMrepo (a data repository for Gut Microbiota); and 20 samples, 10 from CRC patients and 10 from healthy controls, were randomly selected from samples contained in the PRJNA397219 project. Then, Kneaddata software (http://huttenhower.sph.harvard.edu/kneaddata) was utilized for quality controlling and de-hosting of data sequencing on the basis of the human_genome bowtie2 database, MetaPhlAn2 software (http://huttenhower.sph.harvard.edu/metaphlan2) was used to analyze the species composition of intestinal microbiota, and HUMAnN2 software (http://huttenhower.sph.harvard.edu/humann2) was employed to annotate microbial species and functional genes. After that, a differential analysis of the relative abundance of healthy samples and CRC samples was performed using linear discriminant analysis (LDA) effect size (LEfSe) method, with LDA values > 2.0 and *p* < 0.05 considered as significant [26]. Further based on a previously reported spectrum of fecal metabolites in colorectum [27], differential metabolites between CRC and healthy samples were identified using Limma package in R language, with log2FC > 0.4 and *p* < 0.05 as the screening threshold. Metabolic signaling pathways associated with the differential metabolites were revealed utilizing the MetaboAnalyst5.0 tool (https://www.metaboanalyst.ca/). The aforementioned analyses were performed under 3.4.3 R language and Python 2.7 programming language.

### Clinical specimen collection

Tumor tissues and adjacent normal tissues were collected from 83 patients (47 males and 36 females, ranging from 15 to 82 years old with a median age of 58 years) who were pathologically diagnosed with CRC and underwent surgical treatment in Rong chang District People's Hospital of Chongqing from June 2012 to August 2014. None of these patients had received chemotherapy, radiotherapy, or biotherapy before operation, and they were followed up until December 30, 2019. The tumor staging was performed according to AJCC TNM Staging Standard Edition 6 [28]. The degrees of tumor differentiation, including undifferentiated, poorly differentiated, moderately differentiated, and well differentiated adenocarcinoma, were identified according to WHO Classification Standard [29]. All pathological diagnoses [30] were confirmed by two senior pathologists. The tissues were fixed with 10% neutral formalin, embedded in paraffin, and sectioned at 4 μm.

Moreover, the follow-up lasting for 60 months or ended with patients’ death, and their survival was analyzed using Kaplan-Meier method. The time interval between the operation and the death was defined as the overall survival (OS).

### Immunohistochemical staining (IHC)

The slices were incubated in a pressure cooker with 0.01 M citric acid buffer (pH 6.0) for 30 min for antigen retrieval and then treated with 3% hydrogen peroxide for 5 min. Next, the slices were incubated overnight with primary antibodies, including rabbit anti-human SIRT1 (ab189494, 1:500, Abcam, Cambridge, UK), rabbit anti-human β-catenin (ab32572, 1:500, Abcam), rabbit anti-mouse β-catenin (ab16051, 1:500, Abcam), and rabbit-anti human FOXQ1 (ab51340, 1:200, Abcam) at 4°C. Diaminobenzidine (DAB) was then used for immunostaining according to the manufacturer’s instructions. The negative control (NC) group was treated with isotypic antibody. The slices were then observed under a microscope, and at least 10 high-power fields of view were selected per slice for counting of immunostained cells. The expression of SIRT1, β-catenin and FOXQ1 was semi-quantitatively scored according to the staining intensity and distribution. The samples were divided into high-expression group and low-expression group according to the median expression of FOXQ1.

### Cell culture

A normal human colonic epithelial cell line NCM460 (CC-Y1550, Shanghai EK-Bioscience Biotechnology Co., Ltd., Shanghai, China) and 4 CRC cell lines including HCT116 (CCL-247, American type culture collection [ATCC]), DLD-1 (CCL-22, ATCC), HT29 (HTB-38, ATCC) and LoVo (CCL-229, ATCC) were cultured under 37°C and 5% CO_2_ in RPMI 1640 medium (31870082, Gibco, Carlsbad, CA) supplemented with 10% FBS (26140079, Gibco) and penicillin streptomycin (100 U/mL). HEK-293T cells (ACS-4500, ATCC) were cultured in DMEM medium (11995123, Gibco) supplemented with 10% FBS and 1% penicillin-streptomycin (11995123, Gibco). The culture medium was renewed every 24 h, and the cells were dissociated with 0.25% trypsin every 72 h for sub-culture. Further, upon cell confluence reaching 70 ~ 80%, the cells in logarithmic growth phase were used for the following experiments. qRT-PCR was used to screen out the CRC cell lines with high or low FOXQ1 expression for the following experiments.

### Development of radiation-resistant cell sublines

The CRC cell lines HCT116 and HT29 in logarithmic growth phase and good condition were irradiated by X-ray machines/generators (tube voltage of 150 KV, tube current of 20 mA, 7.180 Gy/m at the probe position, and 4.81 Gy/m of the irradiated object). The distance from the focus to the specimen was 350 mm and the first dose was 1 Gy. After each X-ray irradiation, the medium was renewed for further incubation. When the cell confluence reached 90%, the cells were sub-cultured. When the cells entered the logarithmic growth phase again, they were subjected to irradiation at the order of 1 Gy three times, 2 Gy 3 times, and 4 Gy 7 times, followed by screening of radiation-resistant cells. The CRC cells exhibiting resistance to radiation were named HCT116R and HT29R cells.

### Cell transfection and grouping

When the confluence of cells reached 80-90%, transfection was performed according to the protocols of Lipofectamin 2000 (11668-019, Invitrogen, Carlsbad, CA, USA). All plasmids and sequences were designed and constructed by RiboBio (Guangzhou, China). Three different short hairpin RNAs (shRNAs) targeting FOXQ1, i.e., sh-FOXQ1#1, sh-FOXQ1#2 and sh-FOXQ1#3 were prepared (Supplementary Table 1), and their knockdown efficiency was verified by Western blot. Cell grouping of HCT116R and HT29R cells according to different transfections was described as follows.

For the first set of groups, HCT116R cells were transduced with plasmids carrying shRNA targeting FOXQ1 (sh-FOXQ1 group) or corresponding NCs (sh-NC group), and HT29R cells were transduced with FOXQ1 overexpression plasmids (labeled as oe-FOXQ1 group) or corresponding NCs (oe-NC group).

For the second set of groups, HCT116R and HT29R cells were transduced with plasmids carrying shRNA targeting FOXQ1 alone or SIRT1 overexpression plasmids alone or their combination, or corresponding NCs, labeled as sh-FOXQ1 + oe-NC, sh-NC + oe-SIRT1, sh-FOXQ1 + oe-SIRT1, and sh-NC + oe-NC groups.

For the third set of groups, HCT116R cells were treated with EX527, a specific inhibitor for SIRT1 (labeled as EX527 group), or DMSO (DMSO group), and HT29R cells were transduced with SIRT1 overexpression plasmid (oe-SIRT1 group) or corresponding NCs (oe-NC group).

For the fourth set of groups, HCT116R and HT29R cells were transduced with β-catenin overexpression plasmids alone or in combination with plasmids carrying shRNA targeting FOXQ1/ EX527 treatment, or corresponding controls (empty vector or DMSO), labeled as sh-NC + oe-β-catenin, sh-FOXQ1 + oe-β-catenin, Vector, EX527 + oe-β-catenin, and DMSO + oe-β-catenin groups.

### Sphere formation assay

In 2 ml sphere forming medium, the single cell suspension (1000 cells in total) was seeded into the ultra-low attachment pore of Costar 6-well plates (Corning Inc., Corning, NY). After 7 days of culture, formed spheres were observed through microscopy.

### MTT assay

The cells were seeded into 6-well plates with a density of 5 × 10^3^ cells/well and cultured for 14 ~ 16 h. Cells in each group were treated with X-ray (0 Gy, 2 Gy, 4 Gy, 6 Gy, 8 Gy). Untreated cells were used for control. After 72 h, the cells were washed with phosphate-buffered saline (PBS), then treated with 0.5 mg/ml of MTT reagent (Sigma, St Louis, MO), and incubated at 37°C for 4 h. Subsequently, the supernatant was removed and 150 μL DMSO was added. The optical density was examined at 570 nm and cell viability was analyzed.

### Colony formation assay

The cells in logarithmic growth phase were collected, digested with trypsin, centrifuged, and resuspended with DMEM medium; the cell suspension was seeded into 6-well cell culture plates (5 × 10^2^ cells/per well) and each group was repeated in three wells. Allowed to stand for 20 min on the laminar flow bench, the cells were cultured for 14 days and then fixed with 10% paraformaldehyde for 5 min, and stained with 1% crystal violet staining solution for 10 min, followed by counting of colonies formed under an inverted microscope (MI12, MSHOT, Guangzhou, Guangdong, China). Colony formation rate = colony number/seeded cell number × 100%.

### Transwell assay

The invasive ability of CRC cells was detected using Transwell assay. Matrigel (BD, Franklin Lakes, NJ) was diluted in serum-free medium at the ratio of 1:9, then added to the bottom of the upper chamber of 8 μm Transwell system (Corelle, Kenny Bunker), and cultured at 37°C for 2h. HCT116R and HT29R cells were pretreated with serum-free medium for 24 h. The serum-free medium was added into the upper chamber and the complete medium was added to the lower chamber. After 24 h of culture, the cells were fixed with methanol and stained with 0.1% crystal violet solution. The cells were observed with a DMI4000B microscope (Leica, Wetzlar, Germany), images were photographed, and 5 fields of view were randomly selected for cell counting.

### Flow cytometry

Flow cytometry was conducted to detect cell apoptosis. Briefly, 100 μL 1 × Annexin V binding solution was used to prepare cell suspensions, which were then fixed with 75% alcohol for 30 min and subjected to subsequent staining of 5μL AnnexinV-FIOV and 5μL propidium iodide (PI). Then, the cells were cultured in dark at room temperature for 15 min, and 400 μL 1 × Annexin V binding solution was added before testing.

### Real-time quantitative reverse transcription polymerase chain reaction (qRT-PCR)

Total RNA was extracted from cell lines and frozen tissue specimens using TRIzol® Reagent (15596-018, Beijing Solarbio Technology Co., Ltd., Beijing, China) in accordance with the manufacturer’s instructions. PrimeScript™ RT-PCR kit (TaKaRa, Mountain View, CA) was used to reversely transcribe RNA into cDNA to. SYBR Premix Ex Taq^TM^ (TaKaRa) was used to perform qRT-PCR on lightcycle 480 system (Roche Diagnostics, Pleasanton, CA). GAPDH was used as human internal reference and β-actin as murine internal reference, to which the expression of mRNA was normalized. The primers for amplification were designed and purchased by Shanghai General Biotechnology Co., Ltd. The primer sequences are shown in Supplementary Table 1. The relative transcription level of the target gene was calculated by the relative quantitative method (2^-△△CT^ method) as previously described [31].

### Western blot

Cells and frozen tissue samples were lysed with RIPA lysis buffer (R0010, Solarbio, China) supplemented with protease inhibitors (Roche). BCA Kit (Pierce, Rockford, IL) was used to determine the protein concentration of each sample. The total protein (30 ~ 50 μ g) was added into loading buffer and then was separated by 10% sodium dodecyl sulfate polyacrylamide gel electrophoresis (SDS-PAGE) and transferred to polyvinylidene fluoride (PVDF) membrane (Merck Millipore, Billerica, MA). The membrane was blocked with 5% skimmed milk for 1 h, and then incubated overnight in a specific monoclonal antibody shaker at 4 °C. After the membrane was washed with TBST, the membrane was incubated with the corresponding secondary antibody for 1 h. The specific protein band was detected with the super signal West Pico chemiluminescence substrate (Thermo Fisher, Waltham, MA). GAPDH antibody was used as the internal reference. The relative expression of the target protein was evaluated by comparing the gray value of the target protein band to that of the internal reference protein band. The main primary antibodies were as follows: rabbit anti human FOXQ1 (ab51340, 1:2000), rabbit anti mouse FOXQ1 (DF13639, 1:1000, Affinity, China), rabbit anti human and mouse SIRT1 (ab189494, 1:1000), rabbit anti human β-catenin (ab32572, 1:5000), rabbit anti mouse β-catenin (ab16051, 1:2000), rabbit anti human and mouse p-β-catenin (ab27798, 1:500), rabbit anti human and mouse Ac β-catenin (#9030, 1:1000, CST), rabbit anti human and mouse cyclin D1 (ab134175, 1:20000); the secondary antibody used was goat anti rabbit IgG (ab6721, 1:5000); and antibody against internal reference GAPDH (ab9485, 1:2500). The above antibodies were purchased from Abcam (Cambridge, UK).

### Dual-luciferase reporter gene assay

The wild type (WT) and mutated (MUT) sequence of SIRT1 promoter region was fragmented from distal end to proximal end and constructed with firefly and Renilla luciferase cDNA into GV354 vector (GeneChem, Shanghai, China), respectively. HEK-293T cells were seeded into 24-well plates at the density of 5 × 10^4^ cells/per well 24 h before transfection, and extracted constructs were then co-transduced with oe-NC/oe-FOXQ1 plasmids into cells, followed by12-h incubation at 37°C. Dual-luciferase reporter analysis was performed according to the manufacturer’s instructions (Promega, Madison, Wisconsin) and the luciferase activity of firefly and Renilla luciferases was evaluated using the dual luciferase reporter assay system (Promega, Madison, Wisconsin). The ratio of firefly luciferase activity to Renilla luciferase activity indicated the relative activity of luciferase.

### TOP/FOP luciferase reporter assay

HCT116R and HT29R cells in logarithmic growth phase were seeded into 96-well at the density of 1 × 10^4^/mL. TOP flash or FOP flash (0.2 μg/well) were co-transduced with 20 ng pRL TK plasmid (internal reference of dual-luciferase reporter system) into HCT116R and HT29R cells in 96-well plates with Lipofectamine 2000 (0.25 μL/well). Later, HCT116R and HT29R cells were treated according to the aforementioned cell grouping. After 24 h, the cells were lysed, and three parallel wells were set for each group. According to the manufacturer’s instructions, luciferase activity was measured using a bispecific luciferase reporter kit (Promega). Results were expressed by TOP/FOP values. Higher ratio reflected stronger activity.

### Immunofluorescence assay

The cells were seeded onto the slide at the density of 1 × 10^5^ cells/mL, fixed with 4% cold paraformaldehyde for 20 min, and treated with 0.2% Triton for 10 min, followed by 30-min incubation with 1% bovine serum albumin (BSA) to block non-specific binding. Then, the cells were incubated overnight at 4°C with rabbit anti-human β-catenin antibody (ab32572, 1:250) and subsequently incubated with Alexa-fluor® 555 goat anti-rabbit IgG secondary antibody (ab150078, 1:200) at room temperature for 2 h. Next, DAPI staining was used to stain the cell nucleus. After washing with double distilled H_2_O (ddH_2_O), the slides were placed on glycerin and observed under a fluorescence microscope, images were photographed, and positive cells were counted.

### ChIP assay

ChIP kit (Millipore Company) was used to study the enrichment of FOXQ1 in SIRT1 promoter region. The cells in logarithmic growth phase of each group or CRC tissues and adjacent normal tissues were added with 1% formaldehyde and fixed at room temperature for 10 min to make DNA cross-link with protein, and the complex was then randomly fragmented by ultrasonication, followed by centrifugation at 4 °C, 13000 rpm. The supernatant was collected and divided into two tubes, which were incubated overnight at 4°C with NC normal mouse IgG antibody (ab18413, Abcam) and specific anti-rabbit antibody against FOXQ1 (ab51340 Abcam), respectively. The endogenous DNA-protein complex was precipitated by Protein Agarose/Sepharose, and the supernatant was pipetted out after a short centrifugation. The non-specific complex was washed and de-crosslinked at 65 °C overnight, and the DNA fragments were extracted and purified by phenol/chloroform. The enrichment level of FOXQ1 was detected by qRT-PCR with IgG as the internal reference.

### Immunoprecipitation (IP) assay

Immunoprecipitation (IP) was used to analyze the acetylation level of β-catenin. The cells were lysed with NP-40 cell lysis buffer containing PMSF protease inhibitor (Thermo Fisher Scientific, Waltham, MA) and deacetylase inhibitor (Beyotime, Shanghai, China). Then, immunoprecipitation was performed with rabbit anti-human FLAG (ab205606, 1:30, Abcam) antibody. In short, 2 μg of the antibody was added to 500 μL of cell lysate, incubated overnight at 4°C, and then incubated with protein A/G agarose beads (Thermo Fisher Scientific) for 3 h. The immune complex was washed with PBS containing PMSF with protease inhibitor for 5 times, then boiled with loading buffer, and analyzed by Western blot.

### *In vivo* xenografts in nude mice

Twenty-four SPF BALB/c nude mice (6 weeks old, weighing 20 - 22 g, purchased from Vital River Laboratories, Beijing, China) were utilized for the establishment of xenografts of CRC cells. All nude mice were raised in SPF animal laboratory and caged separately. The laboratory humidity was 60 - 65%, and the temperature was 22 - 25°C. The mice were fed with free food and water under 12-h light/dark cycle. After one week of acclimatization, the health status of nude mice was observed before the experiment.

HCT116R cells were transduced with β-catenin overexpression plasmids alone or in combination with plasmids carrying shRNA targeting FOXQ1, or corresponding NCs, labeled as sh-NC + oe-β-catenin, sh-FOXQ1 + oe-β-catenin, and sh-NC + oe-NC groups. Stably transduced cells were then screened out and suspended.

The nude mice were divided into three groups with 8 mice in each group. The cell suspension concentration of each group was adjusted to 1 × 10^7^/mL. Then, 0.2 mL cell suspension was inoculated subcutaneously into the right groin of each nude mouse. When the volume of the subcutaneous xenograft reached 50 mm^3^, the xenograft was irradiated with 2 Gy X-ray at room temperature for 20 min (twice a week for 5 weeks, and lead shielding was used to avoid radiation damage). After feeding for 5 weeks, the feces of nude mice were collected using cotton swabs following abdominal massage, and collected feces were then subjected to qRT-PCR analysis of the content of intestinal microbiota. After that, the mice were euthanized by intraperitoneal injection of 40 mg/kg pentobarbital (P3761, SIGMA, St. Louis). Tumor tissues were isolated and the mRNA expression of FOXQ1, SIRT1, β-catenin, CD133, SOX2 and OCT4 was determined using qRT-PCR. Western blot assay was used to determine the protein expression of Cyclin D1. The protein expression of β-catenin was detected by IHC staining. The tumor growth curve was drawn at 7, 14, 21, 28 and 35 days. The short diameter (a) and long diameter (b) of the tumor were measured with vernier caliper. The tumor volume was calculated according to the formula π (a^2^b)/6, and the tumor mass was weighed with a balance.

### Statistical analysis

SPSS 21.0 (IBM, Armonk, NY) was used to analyze the research data. The measurement data were expressed as mean ± standard deviation. Unpaired t test was adopted for comparison between two groups, one-way analysis of variance (ANOVA) or repeated measurement ANOVA was used for comparison among multiple groups, and Tukey’s test was selected for pairwise comparison within the group. The nominal data were analyzed through chi-square test, correlation analysis was carried out by Pearson or Spearman method, and the survival curves were tested using Log-rank method. The results were considered statistically significant when *p* < 0.05.

## Results

### FOXQ1 overexpression occurred in CRC samples in CRC-related microarray datasets

**Fig. 1:**
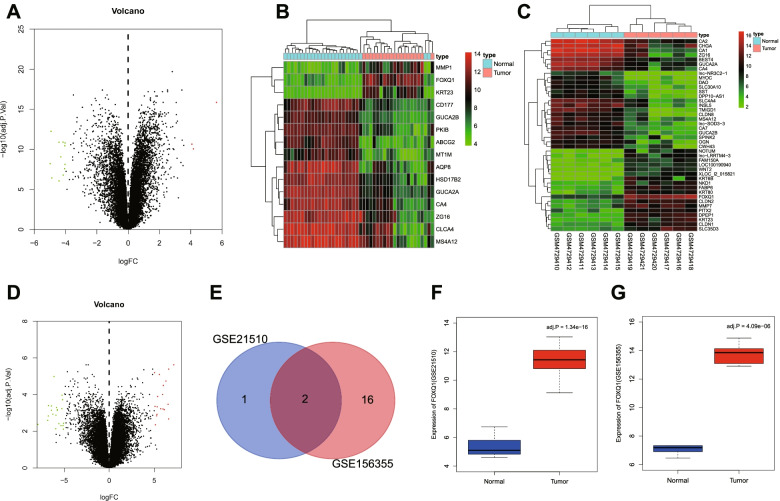
Identification of differentially expressed genes in CRC. **A-B** The volcano plot (**A**) and heatmap (**B**) for differentially expressed mRNAs in CRC samples in CRC-related GSE21510 microarrays; **C-D** The heatmap (**C**) and volcano (**D**) plot for differentially expressed mRNAs in CRC samples in CRC-related GSE156355 microarrays; **E** Candidate CRC-related genes with up-regulated expression in CRC samples (the blue circle indicates those screened out from the GSE21510 microarray and the red circle indicates those from the GSE156355 microarray); **F-G** The expression of FOXQ1 in CRC tissues and adjacent normal samples in GSE21510 and GSE156355 microarrays (red represents CRC samples and blue represents normal samples)

Through bioinformatics analysis based on CRC-related microarray datasets (GSE21510 and GSE156355) retrieved from GEO database, we identified three differentially expressed genes (DEGs) of up-regulated expression and 12 DEGs of down-regulated expression in CRC tissue samples in GSE21510 microarray (Fig. 1A, B) as well as 18 DEGs of up-regulated expression and 24 DEGs of down-regulated expression in CRC tissue samples in GSE156355 microarray (Fig. 1C, D). Further, we found through intersection between these DEGs that only FOXQ1 and KRT23 presented in both of the two CRC-related microarrays (Fig. 1E). Moreover, the expression of FOXQ1 in CRC tissue samples of GSE21510 and GSE156355 datasets was obviously higher than that in normal tissue samples (Fig. 1F, G). Herein, we focused on FOXQ1 in the present study based on our initial finding of FOXQ1 overexpression occurred in CRC samples in CRC-related microarray datasets.

### FOXQ1 was highly expressed in CRC tissues and cells and was positively correlated with the poor prognosis of CRC patients

**Fig. 2:**
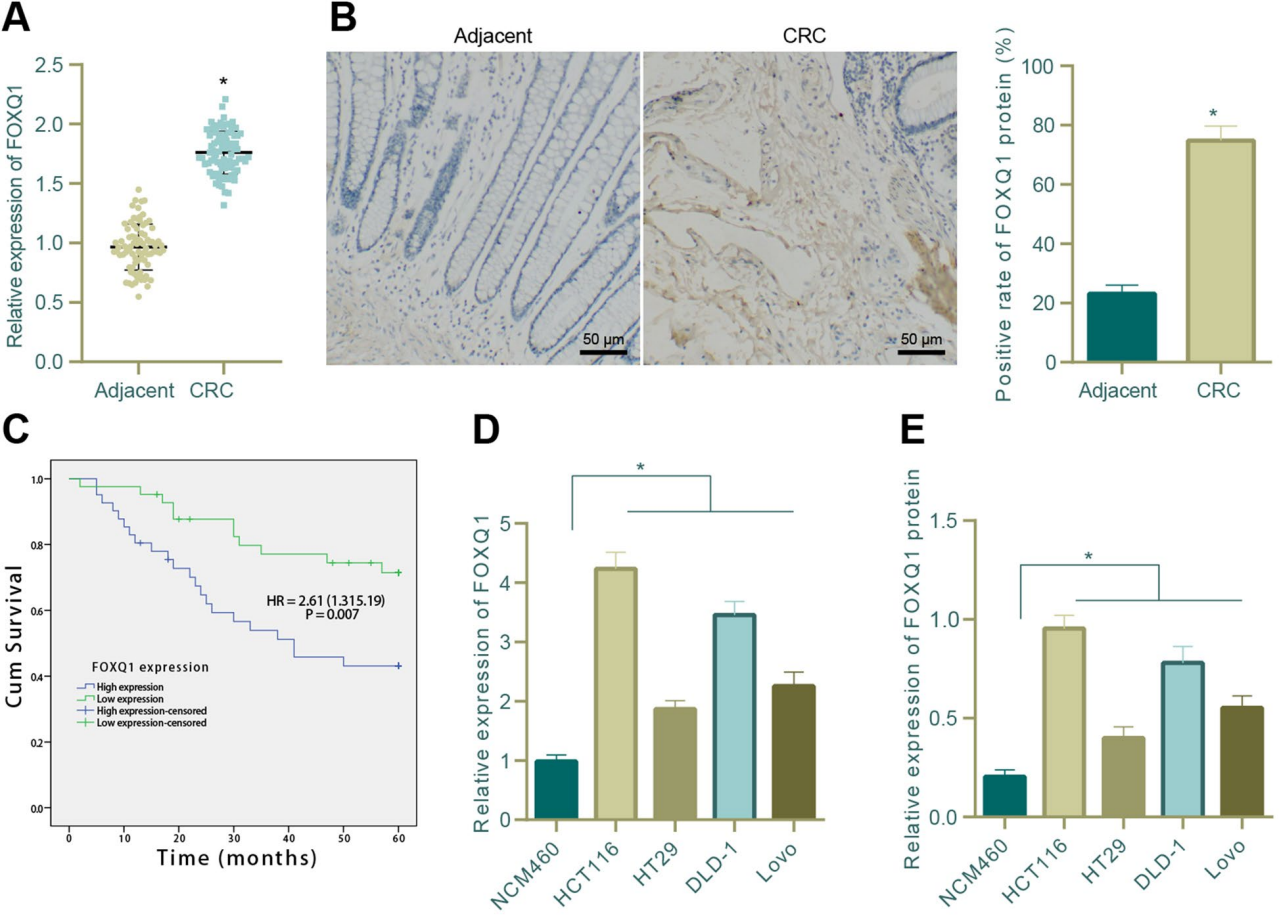
Expression of FOXQ1 in CRC tissues and cells and its correlation with the prognosis of CRC patients. **A** qRT-PCR was used to determine the mRNA expression of FOXQ1 in clinically collected CRC tissues and adjacent normal tissues (*n* = 83, * *p* < 0.05 compared with adjacent normal tissues); **B** IHC was used to determine the protein expression of FOXQ1 in clinically collected CRC tissues and adjacent normal tissues (*n* = 83, * *p* < 0.05 compared with adjacent normal tissues); **C** Kaplan-Meier method was used to analyze the correlation between FOXQ1 protein expression and the overall prognostic survival of CRC patients (*n* = 83); **D** qRT-PCR was used to determine the mRNA expression of FOXQ1 in the normal human colonic epithelial cell line NCM460 and 4 CRC cell lines (* *p* < 0.05 compared with NCM460 cells); **E** Western blot assay was used to measure the protein expression of FOXQ1 in the normal human colonic epithelial cell line NCM460 and 4 CRC cell lines (* *p* < 0.05 compared with NCM460 cells). All cell experiments were repeated for three times independently. The measurement data were expressed as mean ± standard deviation. Unpaired t test was adopted for comparison between groups, one-way ANOVA was used for comparison among multiple groups, and Tukey’s test was selected for pairwise comparison within the group. The survival curve was tested by Log-rank method.

Following bioinformatics analysis, we then explored the expression of FOXQ1 in clinical CRC tissues and CRC cells. Results of qRT-PCR and IHC indicated that the mRNA and protein expression levels of FOXQ1 in clinically collected CRC tissues were elevated as compared with that in adjacent normal tissues (Fig. 2A, B). Then, we conducted the correlation analysis of FOXQ1 protein expression and the clinicopathologic features of 83 CRC patients and found that FOXQ1 protein expression showed no correlation with patient age and gender but a strong correlation with tumor size, TNM staging and lymphatic metastasis (Supplementary Table 2). Based on survival analysis using Kaplan-Meier method, it was revelaed that the OS of CRC patients with low expression of FOXQ1 was significantly longer than those with high expression of FOXQ1 (Fig. 2C).

We further determined the expression of FOXQ1 in 4 human CRC cell lines (HCT116, DLD-1, HT29 and LoVo) and the normal human colonic epithelial cell line NCM460, and the results showed that compared with NCM460 cell line, the mRNA and protein expression levels of FOXQ1 in those 4 CRC cell lines were up-regulated, of which HCT116 cell line exhibited the highest expression of FOXQ1 while HT29 cell line exhibited the lowest expression of FOXQ1 (Fig. 2D, E). Therefore, HCT116 and HT29 cell lines were used for the following experiment.

Collectively, the above results showed that FOXQ1 was highly expressed in CRC tissues and cells and was positively correlated with the poor prognosis of CRC patients.

### FOXQ1 overexpression was observed in radio-resistant CRC cells

**Fig. 3:**
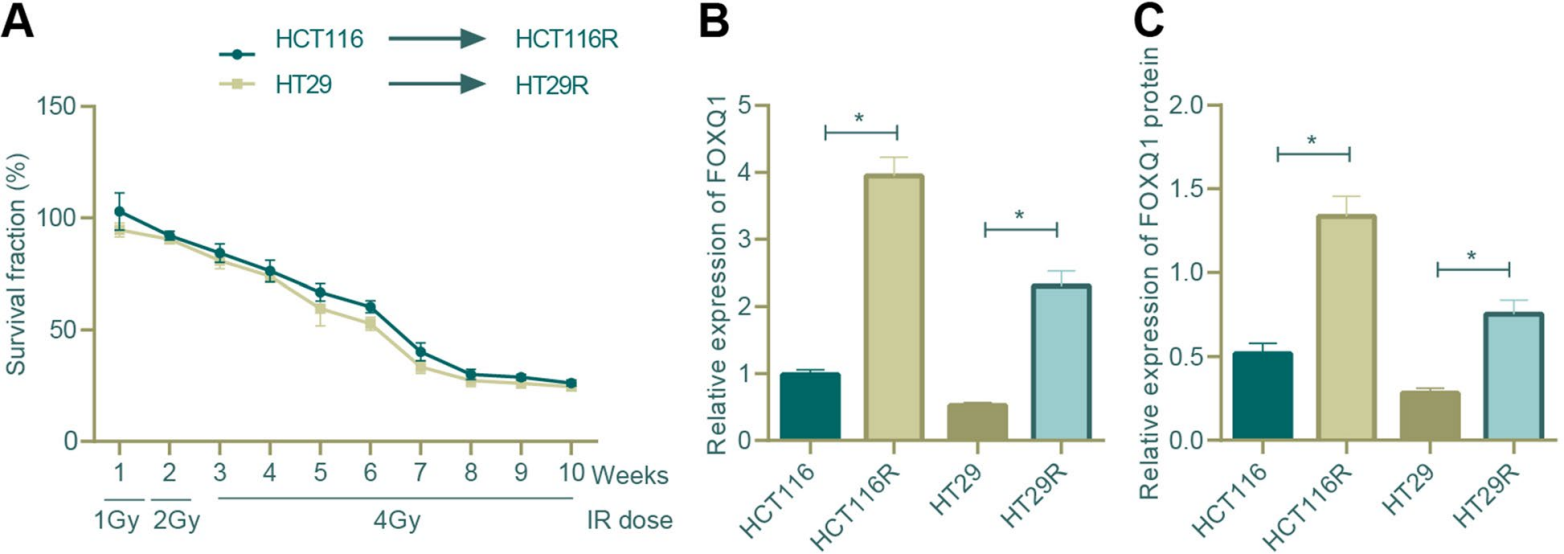
Analysis of the correlation between FOXQ1 expression and CRC cell radio-resistance. **A** The survival rate of HCT116 and HT29 cells during fractionated irradiation to develop radiation-resistant CRC cells; **B** qRT-PCR was used to measure the mRNA expression of FOXQ1 in parent strains HCT116 and HT29 and the radiation-resistant strains HCT116R and HT29R (* *p* < 0.05 between two groups); **C** Western blot assay was used to measure the protein expression of FOXQ1 in parent strains HCT116 and HT29 and radiation-resistant strains HCT116R and HT29R (* *p* < 0.05 between two groups). All cell experiments were repeated for three times independently. The measurement data were expressed as mean ± standard deviation. One-way ANOVA was used for comparison among multiple groups, and Tukey’s test was selected for pairwise comparison within the group.

Shifting to investigation of the potential correlation between FOXQ1 and CRC cell radio-resistance, we developed radio-resistant CRC cells by treating HCT116 and HT29 cells at logarithmic phase with a gradually increasing dose of irradiation (1 Gy three times at the first week, 2 Gy three times at the second week and 4 Gy once a week for the subsequent seven weeks). The survival rate of the cells during irradiation period was examined (Fig. 3A), and 20% of the treated cells were consequently rendered radio-resistant, which were named as HCT116R and HT29R cells. The radio-resistance was further confirmed with colony formation assay, wherein HCT116R and HT29R cells presented an obviously enhanced colony formation ability relative to HCT116 and HT29 cells (Supplementary Fig. 1).

Then, we determined the expression of FOXQ1 in HCT116 and HT29 cells as well as corresponding radiation-resistant HCT116R and HT29R cells, and the results displayed an elevated expression level of FOXQ1 in HCT116R and HT29R cells as compared with HCT116 and HT29 cell (Fig. 3B, C). In this sense, our data illuminated that FOXQ1 overexpression was correlated with CRC cell radiation resistance.

### FOXQ1 knockdown inhibited the stemness and reversed the radio-resistance of CRC cells

**Fig. 4:**
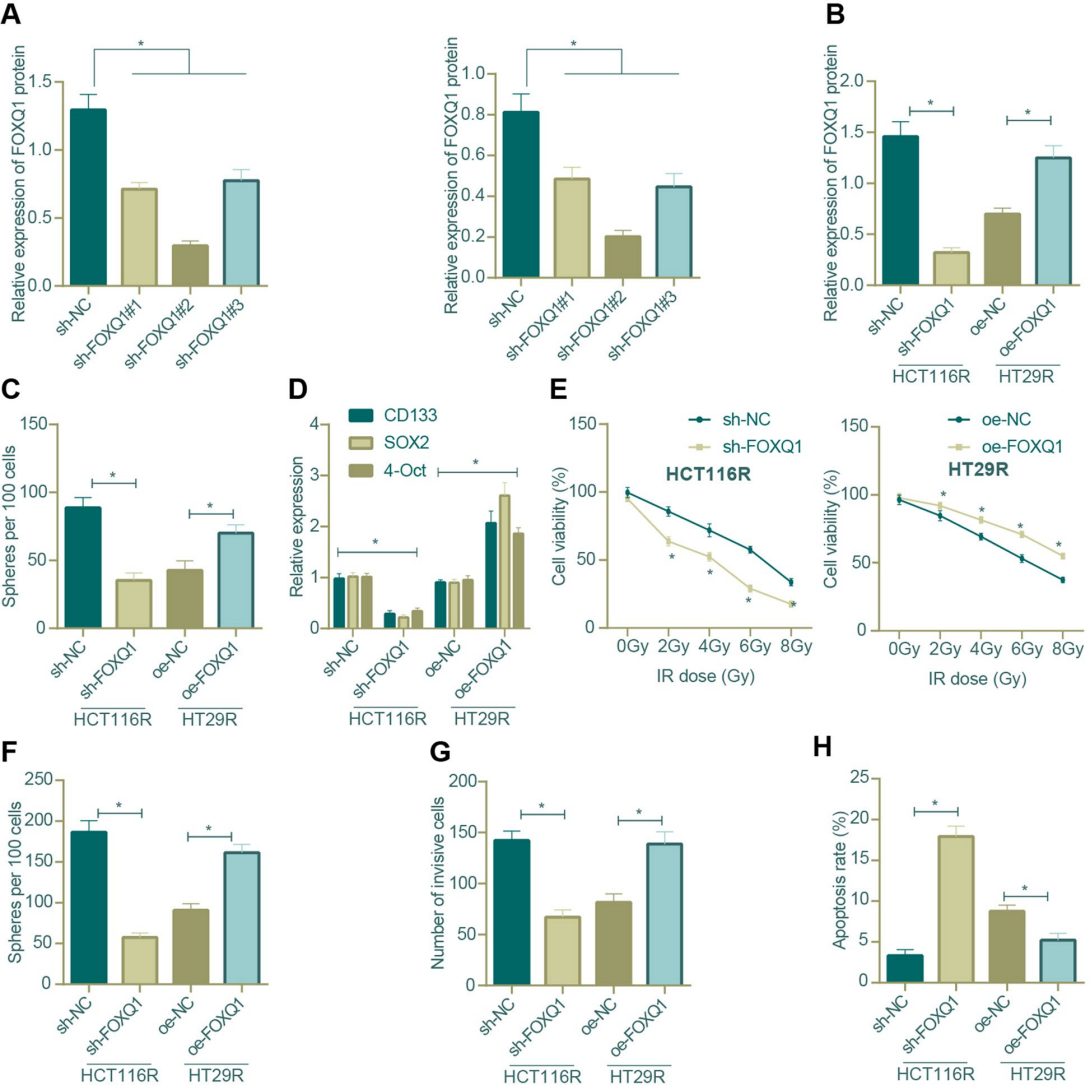
Impact of FOXQ1 knockdown on the stemness and radio-resistance of CRC cells. **A** Western blot assay was used to screen out the most efficient sequence of FOXQ1 knockdown in HCT116R and HT29R cells; **B** Western blot assay was used to determine the changes in the protein expression of FOXQ1 after the treatment of sh-FOXQ1 and oe-FOXQ1 in HCT116R and HT29R cells; **C** The sphere formation assay was carried out to test the tumor sphere formation ability of HCT116R and HT29R cells in the presence of FOXQ1 knockdown/overexpression; **D** qRT-PCR was used to measure the mRNA expression of tumor stem cell markers (CD133, SOX2 and OCT4) in HCT116R and HT29R cells in the presence of FOXQ1 knockdown/overexpression; **E** MTT assay was carried out to test the cell viability of HCT116R and HT29R cells in response to FOXQ1 knockdown/overexpression (* *p* < 0.05 versus sh-NC-treated cells); **F** The colony formation assay to detect the colony formation ability of HCT116R and HT29R cells in response to FOXQ1 knockdown/overexpression; **G** Transwell assay to test the invasion ability of HCT116R and HT29R cells in response to FOXQ1 knockdown/overexpression; **H** Flow cytometry to detect the cell apoptosis of HCT116R and HT29R cells in response to FOXQ1 knockdown/overexpression. *means *p* < 0.05 in the comparison between two groups. All cell experiments were repeated for three times independently. The measurement data were expressed as mean ± standard deviation. Unpaired t test was adopted for the comparison between groups, repeated measures ANOVA was adopted for multi-group comparison, and Tukey’s test was selected for pairwise comparison within the group

To further study the relationship between FOXQ1 and the stemness and radiation resistance of CRC cells, we firstly set up 3 shRNA sequences of FOXQ1, and we found from the detection results of Western blot that sh-FOXQ1#2 presented with the most optimal knockdown efficiency in HCT116R and HT29R cells (Fig. 4A), which was thus selected for the following experiment. Next, we kncoked down FOXQ1 in HCT116R cells and overexpressing it in HT29R cells, which were validated by Western blot assay (Fig. 4B).

Subsequently, it was revealed that FOXQ1 knockdown led to inhibition of tumor sphere growth as well as down-regulated expression of stemness markers (CD133, SOX2 and OCT4) in HCT116R cells, whereas FOXQ1 overexpression led to the opposite in HT29R cells (Fig. 4C, D). Moreover, FOXQ1 knockdown attenuated the viability, colony formation and invasion of HCT116R cells and augmented their apoptosis, while overexpressed FOXQ1 enhanced the viability, colony formation and invasion of HT29 cells and inhibited their apoptosis (Fig. 4E, F, G, H). Collectively, the above results demonstrated that FOXQ1 knockdown inhibited the stemness of CRC cells and reversed their resistance to radiation.

### FOXQ1 transcriptionally activated SIRT1 expression in CRC cells

**Fig. 5:**
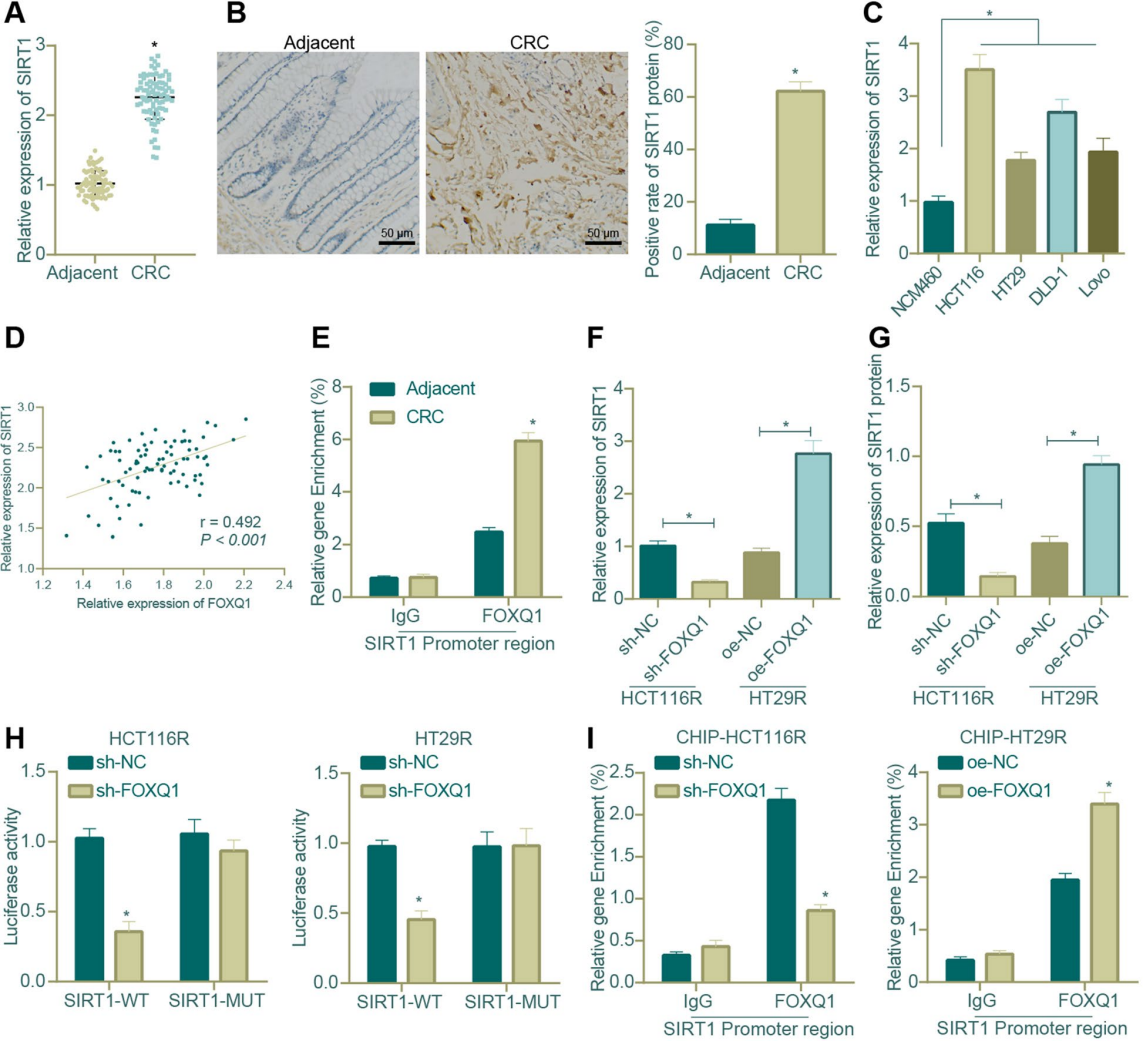
Impact of FOXQ1 on SIRT1 transcriptional expression in CRC. **A** qRT-PCR was used to determine the mRNA expression of SIRT1 in CRC tissues and adjacent normal tissues (*n* = 83; * *p* < 0.05 versus adjacent normal tissues); **B** IHC was used to determine the protein expression of SIRT1 in CRC tissues and adjacent normal tissues (*n* = 83; * *p* < 0.05 versus adjacent normal tissues); **C** qRT-PCR was used to determine the mRNA expression of SIRT1 in the normal colonic human epithelial cell line NCM460 and 4 CRC cell lines (* *p* < 0.05 between two groups); **D** Pearson correlation analysis of SIRT1 and FOXQ1 expression (*n* = 83); **E** ChIP assay was carried out to test the enrichment of FOXQ1 in SIRT1 promoter region in CRC tissues and adjacent normal tissues (*n* = 83; * *p* < 0.05 versus adjacent normal tissues); **F** qRT-PCR was used to measure the SIRT1 mRNA expression in HCT116R and HT29R cells in response to FOXQ1 knockdown/overexpression (* *p* < 0.05 between two groups); **G** Western blot assay was carried out to measure the SIRT1 protein expression in HCT116R and HT29R cells in response to FOXQ1 knockdown/overexpression (* *p* < 0.05 between two groups); **H** The dual-luciferase reporter assay verified the binding of FOXQ1 to SIRT1 promoter region in HCT116R and HT29R cells (* *p* < 0.05 versu sh-NC-treated cells); **I** ChIP assay was carried out to detect the enrichment of FOXQ1 in SIRT1 promoter region after knocking down or overexpressing FOXQ1 in HCT116 and HT29R cells (* *p* < 0.05 verusu cells treated with sh-NC or oe-NC); * *p* < 0.05 in comparison between two groups, and # *p* > 0.05 compared with HT29 cells. All cell experiments were repeated for three times independently. The measurement data were expressed as mean ± standard deviation. Unpaired t test was adopted for the comparison between two groups. The correlation analysis was carried out by Pearson method

Since the aforementioned experiemnts have substantiated the regulatory effects of FOXQ1 on CRC cells, we then explored whether FOXQ1 could regulate SIRT1 expression in CRC. First, we identifiedthat the mRNA and protein levels of SIRT1 in CRC tissues was much higher than that of adjacent normal tissues (Fig. 5A, B); and its mRNA expression was elevated, as compared with that in NCM460 cell line, in the four CRC cell lines, of which HCT116 cell line showed the highest expression of SIRT1, while HT29 cell line showed the lowest (Fig. 5C). We subsequently found through Pearson correlation analysis that there was a significant positive correlation between SIRT1 expression and FOXQ1 expression (Fig. 5D).

Results of ChIP assay then showed that the enrichment level of FOXQ1 in cancer tissues in SIRT1 promoter region was much higher than that in adjacent normal tissues (Fig. 5E). Later, the level of SIRT1 was down-regulated in sh-FOXQ1-treated HCT116R cells and up-regulated in HT29R cells overexpressing FOXQ1 (Fig. 5F, G). Moreover, the results of dual-luciferase reporter assay verified that FOXQ1 knockdown significantly reduced the luciferase activity of SIRT1-wild type (WT), but it did not affect the luciferase activity of SIRT1-mutant (MUT), indicating that FOXQ1 bound to SIRT1 promoter region (Fig. 5H). The results of ChIP assay also found that FOXQ1 was enriched in SIRT1 promoter region in HCT116R and HT29R cells (Fig. 5I). In summary, our results showed that FOXQ1 enhanced SIRT1 transcriptional expression by enrichment in SIRT1 promoter region, thereby increasing the level of SIRT1 in CRC tissues and cells.

### FOXQ1 knockdown down-regulated SIRT1 expression, thereby inhibiting CRC stem cell phenotypes and reversing radio-resistance

**Fig. 6:**
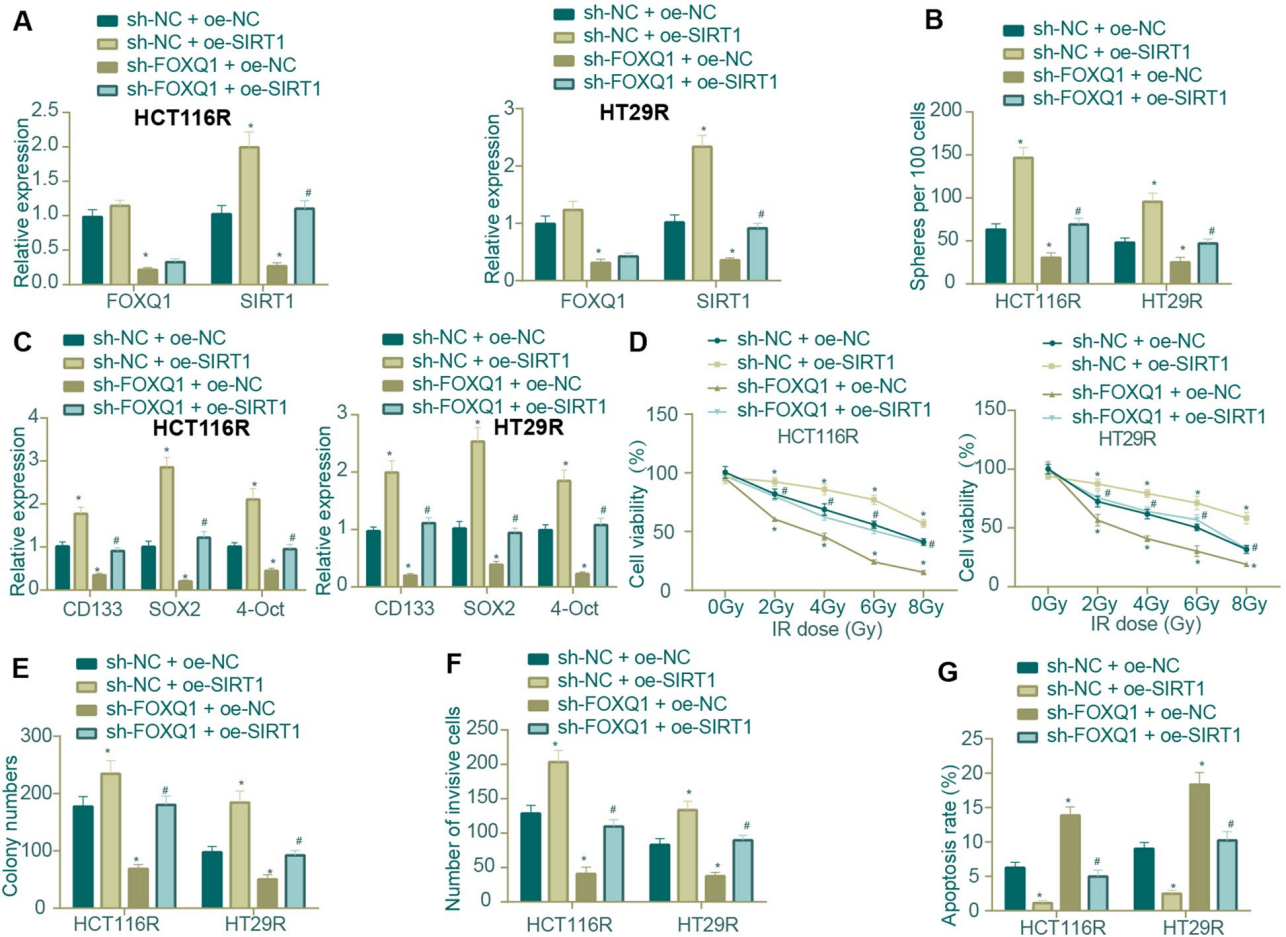
Impact of FOXQ1 knockdown or SIRT1 overexpression on the stemness and radio-resistance of CRC Cells. **A** qRT-PCR was used to determine the mRNA expression levels of FOXQ1 and SIRT1 in HCT116R and HT29R cells in response to SIRT1 overexpression or FOXQ1 knockdown or their combination; **B** Tumor sphere formation assay was carried out to detect the tumor sphere formation ability of HCT116R and HT29R cells in response to SIRT1 overexpression or FOXQ1 knockdown or their combination; **C** qRT-PCR was used to determine the mRNA expression levels of CD133, SOX2 and OCT4 in HCT116R and HT29R cells in response to SIRT1 overexpression or FOXQ1 knockdown or their combination; **D** MTT was carried out to test the viability of HCT116R and HT29R cells in response to SIRT1 overexpression or FOXQ1 knockdown or their combination; **E** Colony formation assay was carried out to test the clone formation ability of HCT116R and HT29R cells in response to SIRT1 overexpression or FOXQ1 knockdown or their combination; **F** Transwell was carried out to test the invasive ability of HCT116R and HT29R cells in response to SIRT1 overexpression or FOXQ1 knockdown or their combination; **G** Flow cytometry was carried out to test the apoptosis of HCT116R and HT29R cells in response to SIRT1 overexpression or FOXQ1 knockdown or their combination. * *p* < 0.05 compared with sh-NC + oe-NC group, # *p* < 0.05 compared with sh-FOXQ1 + oe-NC group. All cell experiments were repeated for three times independently. The measurement data were expressed in mean ± standard deviation. One-way or repeated measurement ANOVA was used for multi-group comparison, and Tukey’s test was selected for pairwise comparison within the group

After identifying SIRT1 as the downstream gene of FOXQ1, we then examined whether FOXQ1 exerted regulatory effects on CRC cell stemness and radio-resistance through SIRT1. In HCT116R and HT29R cells, SIRT1 overexpression alone led to a nearly unchanged level of FOXQ1 and an elevated level of SIRT1, whereas FOXQ1 knockdown led to down-regulated levels of both FOXQ1 and SIRT1. Besides, the down-regulation of SIRT1 caused by FOXQ1 knockdown alone was reversed in response to additional oe-SIRT1 treatment (Fig. 6A).

In addition, the tumor sphere formation ability of HCT116R and HT29R cells was enhanced in response to SIRT1 overexpression and reduced in response to FOXQ1 knockdown. The inhibitory effects of FOXQ1 knockdown on sphere formation was repressed when FOXQ1 knockdown was combined with SIRT1 overexpression (Fig. 6B). Further, we utilized qRT-PCR to determine the mRNA expression levels of tumor stem cell markers (CD133, SOX2 and OCT4) in CRC cells and found that their levels were elevated in the presence of SIRT1 restoration and decreased in the presence of FOXQ1 knockdown alone, which could be reversed by the combination of FOXQ1 knockdown and SIRT1 overexpression (Fig. 6C).

The results of MTT, clone formation, and Transwell assays subsequently showed that the viability, colony formation, and invasive ability of HCT116R and HT29R cells were enhanced in response to SIRT1 overexpression but down-regulated in response to FOXQ1 knockdown, whereas their combination reversed the down-regulation caused by FOXQ1 knockdown alone (Fig. 6D-F). Moreover, the enhanced apoptotic potential of HCT116R and HT29R cells by FOXQ1 knockdown alone was repressed when FOXQ1 knockdown was combinaed with SIRT1 overexpression, although still higher than that in response to SIRT1 overexpression alone (Fig. 6G).

Taken together, the aforementioned results substantiated that FOXQ1 knockdown attenuated stemness and radio-resistance in radiation-resistant CRC cells by inhibiting SIRT1 expression, while overexpressed SIRT1 reversed these effects of FOXQ1 knockdown.

### SIRT1 enhanced β-catenin expression and activity, thereby stimulating β-catenin translocation to cell nucleus

**Fig. 7:**
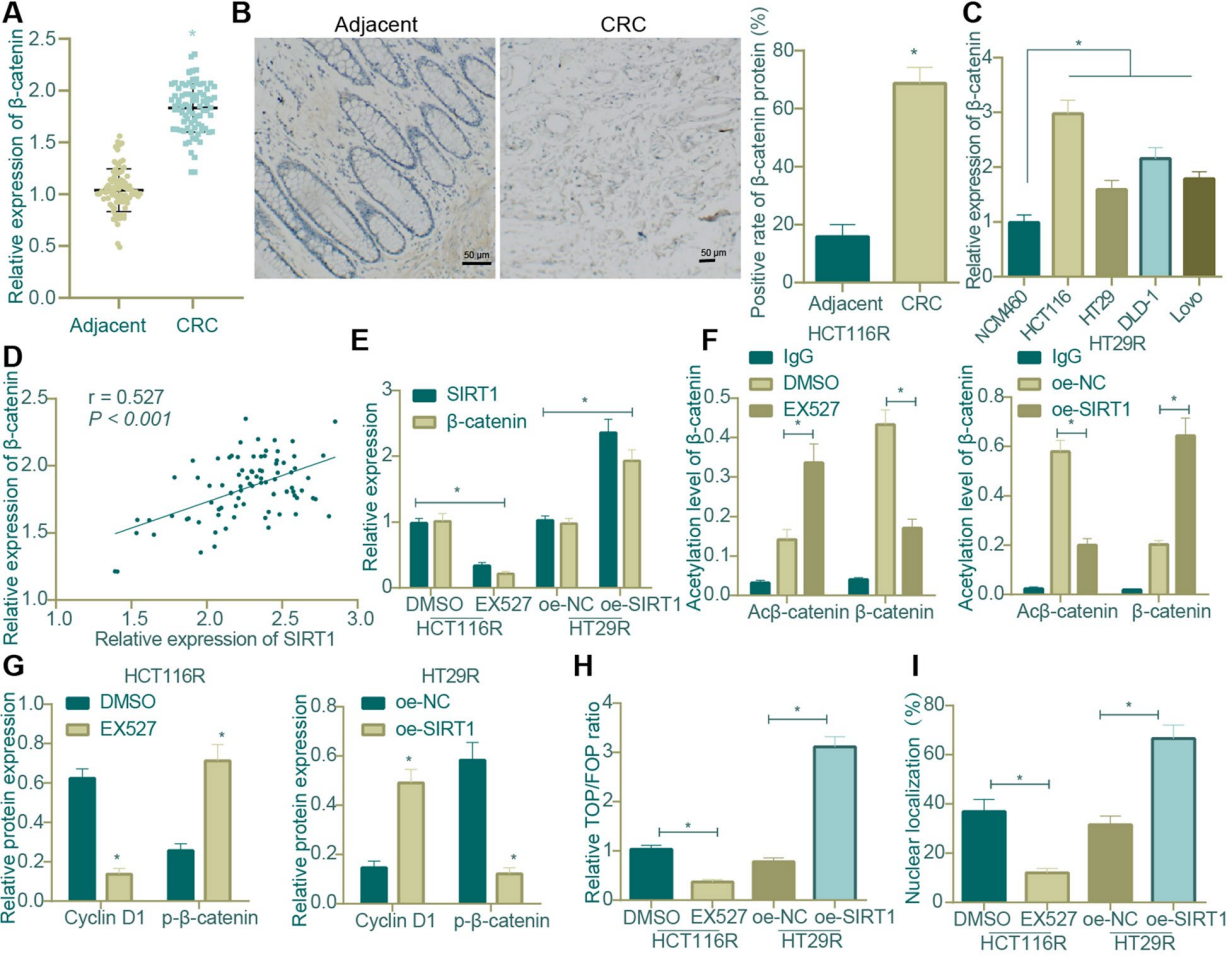
Impact of SIRT1 on nuclear translocation and activity of β-catenin in CRC. **A** qRT-PCR was used to determine the mRNA expression of β-catenin in CRC tissues and adjacent normal tissues (*n* = 83, * *p* < 0.05 compared with adjacent normal tissues); **B** IHC was used to measure the protein expression positive rate of β-catenin in CRC tissues and adjacent normal tissues (*n* = 83, * *p* < 0.05 compared with adjacent normal tissues); **C** qRT-PCR was used to determine the mRNA expression of β-catenin in the normal human colonic epithelial cell line NCM460 and 4 CRC cell lines; **D** Pearson correlation analysis was used to evaluate the correlation between β-catenin and expression of SIRT1 (*n* = 83); **E** qRT-PCR was used to determine the mRNA expression of SIRT1 and β-catenin in HCT116R and HT29R cells in response to SIRT1 overexpression/inhibition (* *p* < 0.05 compared with DMSO or oe-NC); **F** IP assay was carried out to detect the acetylation level of β-catenin in HCT116R and HT29R cells in response to SIRT1 overexpression/inhibition; **G** Western blot assay was used to measure the protein expression of p-β-catenin and Cyclin D1, a downstream target gene of β-catenin, in HCT116R and HT29R cells in response to SIRT1 overexpression/inhibition (*p* < 0.05 verusu cells treated with DMSO or oe-NC); **H** TOP/FOP luciferase reporter was used to detect the luciferase activity of β-catenin in HCT116R and HT29R cells in response to SIRT1 overexpression/inhibition; **I** immunofluorescence was used to detect the nuclear translocation of β-catenin protein in HCT116R and HT29R cells in response to SIRT1 overexpression/inhibition (*p* < 0.05 between two groups). All cell experiments were repeated for three times independently. The measurement data were expressed as mean ± standard deviation. Unpaired t test was adopted for the comparison between groups. Correlation analysis was carried out by Pearson. One-way ANOVA was used for multi-group comparison, and Tukey’s test was selected for pairwise comparison within the group

Since β-catenin is highly expressed in CRC, and SIRT1-mediated deacetylation has been correlated with β-catenin expression [19, 32], we then managed to investigate how SIRT1 regulates β-catenin in CRC. First, we validated through qRT-PCR and IHC that the mRNA and protein expression of β-catenin was up-regulated in CRC tissues versus that in adjacent normal tissues (Fig. 7A-B) and also in CRC cell lines, of which HCT116 cell line exhibited the highest and HT29 cell line exhibited the lowest expression of β-catenin (Fig. 7C). The results of Pearson correlation analysis found a significant positive correlation between the expression of β-catenin and SIRT1 (Fig. 7D).

In addition, inhibition of SIRT1 induced by EX527, a specific and effective SIRT1 inhibitor, resulted in decreased levels of SIRT1 and β-catenin in CRC cells, and SIRT1 restoration led to the opposite (Fig. 7E). Co-IP assay results showed that the level of β-catenin in EX527-treated HCT116R cells was decreased while that of Acβ-catenin was increased; the level of β-catenin in HT29R cells overexpressing SIRT1 was increased while that of Acβ-catenin was decreased (Fig. 7F).

Further, EX527-treated HCT116R cells presented with up-regulated level of p-β-catenin but a down-regulated level of Cyclin D1, a previously documented downstream target gene of β-catenin [33, 34], and the opposite was found in HT29R cells overexpressing SIRT1 (Fig. 7G). Moreover, the activity of β-catenin, measured by TOP/FOP luciferase reporter assay, was shown to be reduced in response to EX527-induced inhibition of SIRT1 and elevated in response to SIRT1 overexpression (Fig. 7H). The results of immunofluorescence assay subsequently revealed that the number of β-catenin-positive HCT116R cells was obviously decreased after EX527 treatment, accompanied by diminished nuclear translocation of β-catenin; in contrast, SIRT1 restoration in HT29R cells led to increased number of β-catenin-positive cells as well as augmented nuclear translocation of β-catenin (Fig. 7I).

In summary, SIRT1 triggered the expression and activity of β-catenin, thereby facilitating the translocation of β-catenin into cell nucleus.

### β-catenin may participate in the urea cycle-related metabolic pathway and affect the intestinal microbiota structure in gut

**Fig. 8:**
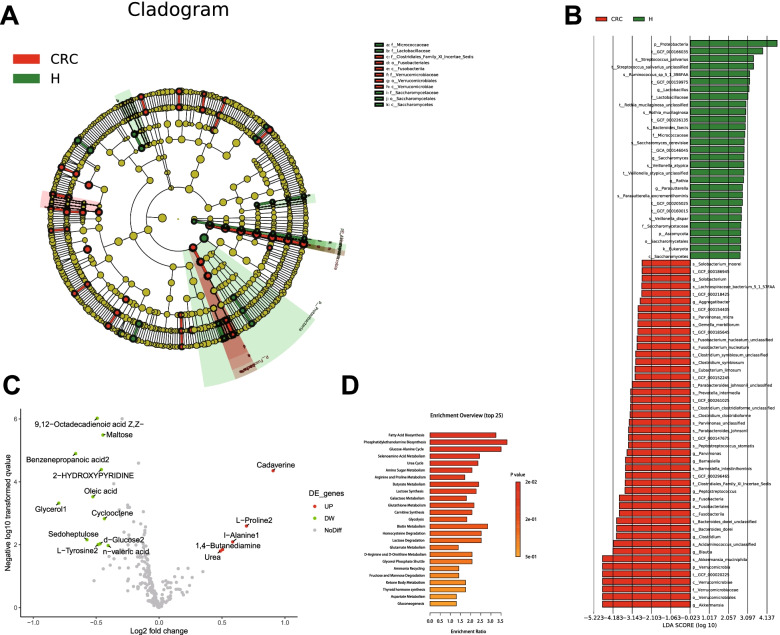
Linear discriminant analysis (LDA) integrated with effect size (LEfSe). **A** Cladogram indicating the phylogenetic distribution of microbiota correlated with the healthy or CRC groups. **B** The differences in abundance between the healthy and CRC groups. **C** Volcano plot of metabolite content in feces samples of the healthy and CRC groups (the color scale from green to red indicates the log2 (FC) (CRC/H) of metabolites from low to high). **D** Results of the metabolite pathway enrichment analysis

Further to examine whether the intestinal microbiota is involved in the resistance of CRC to radiotherapy, we performed a differential analysis of the microbial species in healthy samples and CRC samples using LEfSe method. According to the results, the proportions of several pathogenic bacteria including Akkermansia, Blautia and Clostridium were up-regulated (LDA scores (log10) > 4) in the feces samples of CRC patients, while Lactobacillus, Saccharomyces, Rothia, Parasutterella and faecalis are the most abundant (LDA score (log10)>2) microbiota in healthy samples (Fig. 8A-B). Subsequent analysis of metabolites indicated that Maltose, n-valeric acid, Oleic acid, Sedoheptulose, Urea, 1,4-Butanediamine, Benzenepropanoic acid, Cadaverine and Cyclooctene were of differential content in CRC samples relative to healthy ones (Fig. 8C).

Then, an enrichment analysis of metabolites related to biological functions was performed utilizing the MetaboAnalyst5.0 software. It was revealed that CRC-related intestinal metabolites were mainly involved in biological functions including Fatty Acid Biosynthesis, Phosphatidylethanolamine Biosynthesis, Glucose-Alanine Cycle and Selenoamino Acid Metabolism (Fig. 8D). Furthermore, it was identified through HMDB database that urea (HMDB0000294) and lithium (HMDB0005949) were metabolites related to β-catenin (HMDBP02169), and urea was involved in the urea cycle-related metabolic pathway.

Collectively, these results led to a speculation that β-catenin may participate in the urea cycle-related metabolic pathway and affect the structure of the intestinal microbiota, thereby regulating the stemness and radio-resistance of CRC cells.

### FOXQ1 knockdown inhibited the *in vivo* tumor formation of radio-resistant CRC cells through repressing CRC stemness and related intestinal bacteria *via* blocking the SIRT1/β-catenin axis

**Fig. 9:**
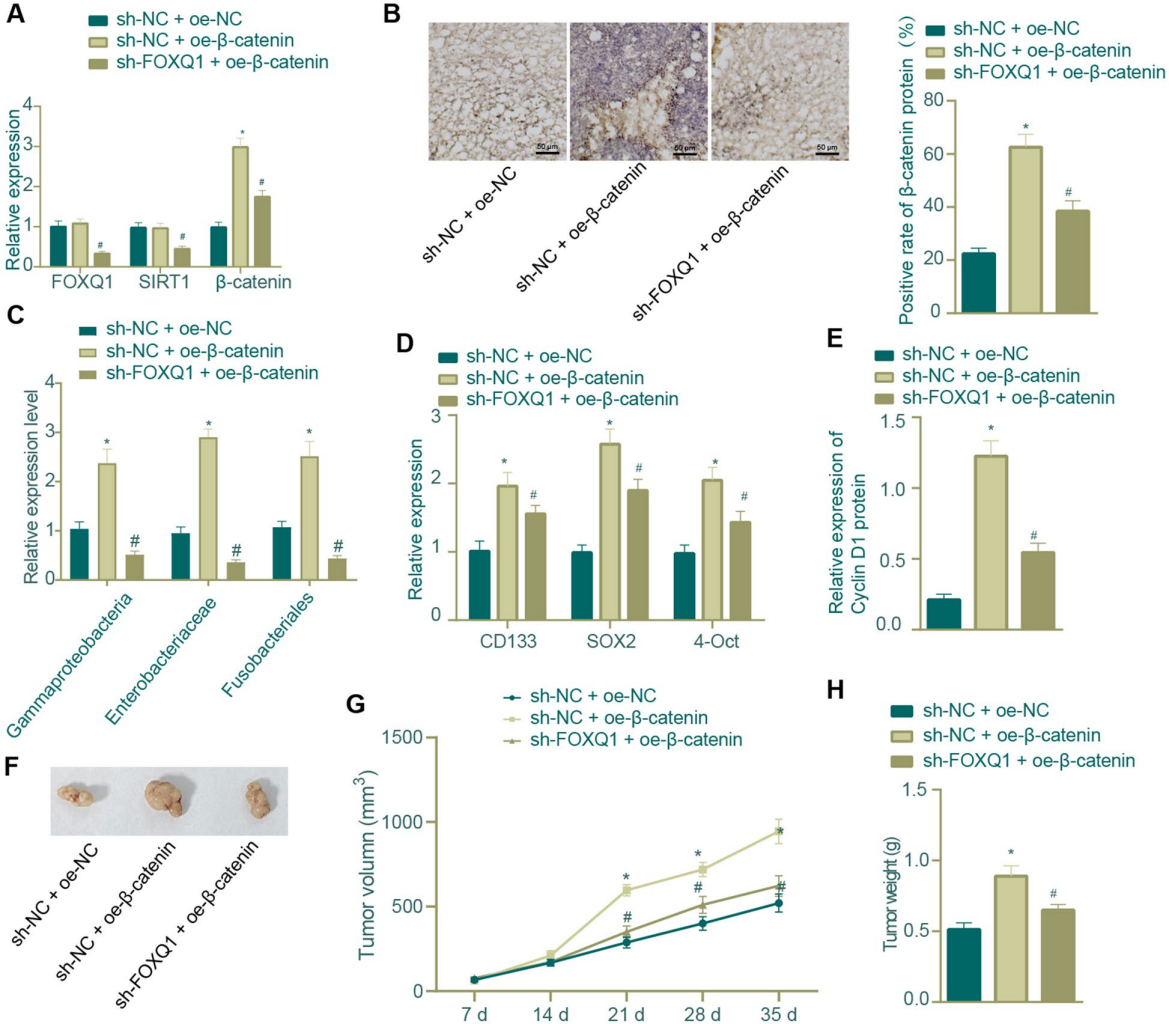
Impact of FOXQ1/SIRT1/β-catenin axis on xenograft formation ability within radiation-resistant CRC cells. **A** qRT-PCR was used to determine the mRNA expression of FOXQ1, SIRT1 and β-catenin in the xenograft tissues of nude mice; **B** IHC was used to measure the protein expression positive rate of β-catenin in the xenograft tissues of nude mice; **C** qRT-PCR measurement of the levels of Gammaproteobacteria, Enterobacteriaceae and Fusobacteriales in the feces of nude mice; **D** qRT-PCR was used to determine the mRNA expression of tumor stem cell markers (CD133, SOX2 and OCT4) in the xenograft tissues of nude mice; **E** Western blot assay was used to determine the protein expression of Cyclin D1 in the xenograft tissues of nude mice; **F** Representative tumor anatomy of nude mice; **G** Broken line graph of volume growth change in the xenograft of nude mice; **H** Weight comparison of xenograft of nude mice; * *p* < 0.05 compared with sh-NC + oe-NC group, # *p* < 0.05 compared with sh-NC + oe-β-catenin group. Each group consists of 8 nude mice. The measurement data were expressed as mean ± standard deviation. One-way or repeated measurement ANOVA was used for multi-group comparison, and Tukey’s test was selected for pairwise comparison within the group

Following the aforementioned *in vitro* experiments, we moved to *in vivo* substantiation of the impact of the FOXQ1/SIRT1/β-catenin axis on xenograft formation ability and radiation resistance of radiation-resistant CRC cells. According to the data, xenograft tissues of mice injected with β-catenin overexpression CRC cells presented with elevated mRNA and protein expression levels of β-catenin and nearly unchanged FOXQ1 and SIRT1 as compared with those of mice injected with NC CRC cells; compared with β-catenin overexpression alone, its combination with FOXQ1 knockdown led to decreased levels of FOXQ1, SIRT1 and β-catenin (Fig. 9A-B).

Feces samples were then collected from CRC mice of each group for qRT-PCR analysis of the intestinal microbiota. The results indicated that the content of Gammaproteobacteria, Enterobacteriaceae, and Fusobacteriales (previously identified as CRC-related pathogenic bacteria) in the feces of CRC mice was up-regulated in response to β-catenin overexpression; and additional FOXQ1 knockdown abrogated the up-regulation induced by β-catenin overexpression (Fig. 9C).

The mRNA expression of tumor stem cell markers (CD133, SOX2 and OCT4) in the CRC xenograft tissues of mice was then found to be up-regulated in the presence of β-catenin overexpression alone, and the up-regulation was reversed when β-catenin overexpression was combined with FOXQ1 knockdown (Fig. 9D). Moreover, our data indicated that the increase in Cyclin D1 expression induced by β-catenin overexpression alone could be abrogated by additional FOXQ1 knockdown (Fig. 9E).

In addition, the xenografts grew rapidly and became heavier in mice injected with CRC cells overexpressing β-catenin as compared with mice injected with NC CRC cells; and the tumor-promoting effects of β-catenin overexpression alone was repressed when combined with FOXQ1 knockdown (Fig. 9F-H).

Collectively, our *in vivo* experiments unraveled that FOXQ1 knockdown regulated the SIRT1/β-catenin axis and thus reduced the content of CRC-related intestinal pathologic bacteria to repress the xenograft formation of radiation-resistant CRC cells.

## Discussion

Radiotherapy is a commonly used multimodal nonsurgical treatment for patients with advanced CRC [35]. However, the cancer stem cell subpopulation displaying intrinsic radio-resistance may survive radiotherapy, and reactivation of proliferation in these can lead to increased risk of poor clinical outcomes [36, 37]. Hence, in this study we focused on the underlying mechanism by which radioresistant CRC cells survive and preserve their aggressive phenotype and found that FOXQ1 may enhance the stemness and radio-resistance of CRC stem cells and ameliorate the aberrant intestinal microbiota through the FOXQ1/SIRT1/β-catenin regulatory axis.

Our finding of FOXQ1 overexpression occurred in CRC samples in CRC-related microarrays indicated the potential regulatory role of FOXQ1 in the development of CRC. Subsequently, we identified that FOXQ1 was highly expressed in CRC tissues and cell lines and was positively correlated with the poor prognosis of CRC patients. Our findings corroborate previous studies that revealed the up-regulated expression of FOXQ1 and its cancer metastasis-promoting effects in CRC [13, 38]. FOXQ1, also known as HFH1, is a member of the FOX gene family and a transcription factor of sequence specificity based on its flanking wings [39]. Kaneda et. al. also found overexpressed FOXQ1 in CRC samples and further revealed that FOXQ1 could enhance tumorigenicity and tumor growth *via* its angiogenic and antiapoptotic functions [40]. Interestingly, FOXQ1 overexpression in pancreatic cancer stem cells has been suggested to promote the resistance of cancer stem cells to radiotherapy [14]. In relation to this, our data illuminated that FOXQ1 overexpression was correlated with CRC cell radio-resistance. Although rarely has FOXQ1 been directly associated with radio-resistance in cancers, a number of studies have indicated the correlation between FOXQ1 and cancer stem cells. For instance, FOXQ1 has been considered as a novel target of breast cancer stem cell inhibition [41]; Han et. al. revealed that miR-4319-mediated inhibition of FOXQ1 repressed the epithelial-mesenchymal transition and prevented cancer stemness of hepatocellular carcinoma [42]. Considering this, we manipulated FOXQ1 expression and substantiated that FOXQ1knockdown inhibited the stemness and reversed the radio-resistance of CRC cells. Importantly, it has been well-established that the aggressive phenotypes of cancer stem cells contributed to cancer radio-resistance [37, 43].

Following the regulatory effects of FOXQ1 on CRC cell stemness and radio-resistance, we further managed to explore its downstream mechanisms. We found that FOXQ1 could promote SIRT1 transcriptional activity by enrichment in SIRT1 promoter region, thereby increasing the level of SIRT1 in CRC tissues and cells. A previous study also indicated that FOXQ1 regulated senescence-associated inflammation through triggering the expression of SIRT1 [15]. The class III deacetylase SIRT1, a member of the sirtuin protein family, plays a critical role in a variety of cancers [44, 45]. Sun et al. observed SIRT1 overexpression in CRC and found that SIRT1 may suppress CRC metastasis [46]. Of note, SIRT1 has been highlighted for regulating radiosensitivity of hepatoma cells [47] and breast cancer cells [48]. Consistent with the evidence existing, our data demonstrated that FOXQ1 knockdown inhibited the stemness of radiation-resistant CRC cells and enhanced their apoptosis by inhibiting SIRT1 expression, whereas overexpressed SIRT1 reversed the effects of FOXQ1 knockdown on radiation-resistant CRC cells. Moreover, we continued to investigate the downstream target of the FOXQ1/SIRT1 axis and found that SIRT1 enhanced the expression and activity of transcription factor β-catenin, thereby facilitating the translocation of β-catenin to cell nucleus in CRC cells. These findings corroborate a previous study where SIRT1 was found to trigger the nuclear translocation and activity of β-catenin [19]. Thereafter, CRC-correlated intestinal bacteria and metabolites were identified in our metagenomics analysis, through which β-catenin was revealed to participate in the urea cycle-related metabolic pathway and affect the intestinal microbiota in CRC. Further, through a series of *in vivo* function assays, we demonstrated that FOXQ1 knockdown inhibited the CRC xenograft formation through repressing CRC-related intestinal bacteria *via* modulation of the SIRT1/β-catenin axis in radiation-resistant CRC cells.

## Conclusions

**Fig. 10:**
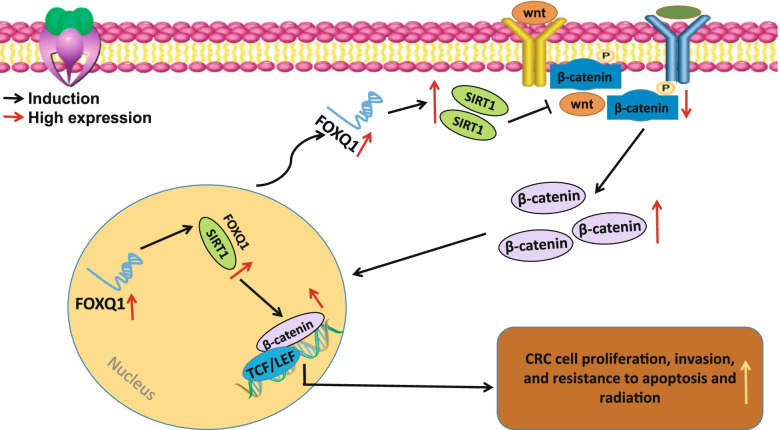
Molecular mechanism graph of the stemness and radio-resistance of CRC cells regulated by the FOXQ1/SIRT1/β-catenin axis. The activation of FOXQ1/SIRT1/β-catenin regulatory circuit may induce CRC stem cell phenotypes, thereby contributing to the resistance of CRC cells to radiotherapy

Taken together, the data acquired in the present study led to a conclusion that FOXQ1 may induce SIRT1 expression, trigger the nuclear translocation and activity of β-catenin, enhance the stemness of CRC cells and benefit CRC-related intestinal pathological bacteria, thereby inducing the resistance of CRC cells to radiation (Fig. 10). Moreover, FOXQ1 knockdown was demonstrated to inhibit the stemness of CRC cells and thus reverse the resistance of CRC cells to radiotherapy. By elucidating the FOXQ1/SIRT1/β-catenin regulatory axis involved in CRC cell radio-resistance, the present study deepened our understanding of the mechanisms related to the radio-resistance in CRC and provided promising strategies for improving the clinical outcomes of radiotherapy for CRC patients.

## Supplementary Information

**Additional file 1: Supplementary Figure 1.** Colony formation assay to detect the colony formation ability of HCT116R and HT29R cells as compared with HCT116 and HT29 cells. * *p* < 0.05 compared with HCT116 cells, # *p* < 0.05 compared with HT29 cells. All cell experiments were repeated for three times independently. The measurement data were expressed as mean ± standard deviation. Unpaired t test was adopted for the comparison between groups.

**Additional file 2: Supplementary Table 1.** Primer Sequences used in qRT-PCR. Note: F, forward; R, reverse; FOXQ1, Forkhead box Q1; SIRT1, sirtuin 1

**Additional file 3: Supplementary Table 2.** Correlation between FOXQ1 expression and clinicopathological features of CRC patients. Note: The data were nominal data, which was analyzed by chi-square test. The sample size (n) was 83, and *p* < 0.05 means that the difference was of statistical significance.

## Data Availability

The data that support the findings of this study are available from the corresponding author upon reasonable request.

## Abbreviations

CRC: Colorectal cancer
FOXQ1: Forkhead box Q1
SIRT1: Sirtuin 1
LDA: Linear discriminant analysis
LEfSe: Linear discriminant analysis effect size
OS: Overall survival
IHC: Immunohistochemical staining
DAB: Diaminobenzidine
NC: Negative control
shRNAs: Short hairpin RNAs
PBS: Phosphate-buffered saline
PI: Propidium iodide
qRT-PCR: Quantitative reverse transcription polymerase chain reaction
SDS-PAGE: Sodium dodecyl sulfate polyacrylamide gel electrophoresis
PVDF: Polyvinylidene fluoride
WT: Wild type
MUT: Mutated
ddH_2_O: Double distilled H_2_O
IP: Immunoprecipitation
ANOVA: Analysis of variance
DEGs: Differentially expressed genes
