# Supplementary material for: FOXQ1-mediated SIRT1 upregulation enhances stemness and radio-resistance of colorectal cancer cells and restores intestinal microbiota function by promoting β-catenin nuclear translocation
Source: J Exp Clin Cancer Res. 2022 Feb 19;41:70. doi: 10.1186/s13046-021-02239-4 (PMC8857837; doi:10.1186/s13046-021-02239-4)
Supplement: Supplementary file 2 — Additional file 2: Supplementary Table 1. Primer Sequences used in qRT-PCR. Note: F, forward; R, reverse; FOXQ1, Forkhead box Q1; SIRT1, sirtuin 1 [file 13046_2021_2239_MOESM2_ESM.docx]

**Supplementary Table 1** **Primer Sequences used in qRT-PCR**

| Gene | Sequence |
| --- | --- |
| sh-FOXQ1#1 | 5′-CGCGGACUUUGCACUUUGA-3′ |
| sh-FOXQ1#2 | 5′-CCAGCTCCTTCGCCATCGACA-3′ |
| sh-FOXQ1#3 | 5′-GGCUGGCUUCAUCCACUGC-3′ |
| sh-NC | 5′-TTCTCCGAACGTGTCACGT-3′ |
| Human FOXQ1-F | 5′-TGATTTCTTGCTATTGACCGATGC-3′ |
| Human FOXQ1-R | 5′-GCCCAAGGAGACCACAGTTAGAG-3′ |
| Human SIRT1-F | 5'-CCGGATTTGAAGAATGTTGG-3' |
| Human SIRT1-R | 5'-ATCTGCTCCTTTGCCACTCT-3' |
| Human β-catenin-F | 5'-TGGTGACAGGGAAGACATCA-3' |
| Human β-catenin-R | 5'-CCATAGTGAAGGCGAACTGC-3' |
| Human CD133-F | 5'-TTCTATGCTGTGTCCTGGGGC-3' |
| Human CD133-R | 5'-TTGTTGGTGCAAGCTCTTCAAGGT-3' |
| Human SOX2-F | 5'-GTCCGAGGCCAGCTCCAGCCCC-3' |
| Human SOX2-R | 5'-GCGGGTTCCGGCACCTCGG-3' |
| Human OCT4-F | 5'-GTGGGGCTCACCCTGGGGGTTC-3' |
| Human OCT4-R | 5'-GCAGAGCCTCAAAGCGGCAGAT-3' |
| Human GAPDH-F | 5′-CTTTGGTATCGTGGAAGGACTC-3′ |
| Human GAPDH-R | 5′-GTAGAGGCAGGGATGATGTTCT-3′ |
| Mouse FOXQ1-F | 5′-AGTAAGGACAGCACTGCACC-3′ |
| Mouse FOXQ1-R | 5′-TTCCAGTCCGGCACAGTTAC-3′ |
| Mouse SIRT1-F | 5′-CTCCACCAGCATTGGGAACT-3′ |
| Mouse SIRT1-R | 5′-GGAAGATGAAGTCAACCAACAGT-3′ |
| Mouse β-catenin-F | 5′-AATCAGCTGGCCTGGTTTGA-3′ |
| Mouse β-catenin-R | 5′-CTGTGGCAAAAACATCAACGTG-3′ |
| Mouse CD133-F | 5′-GGGGTGGTTGTGACCTAGTG-3′ |
| Mouse CD133-R | 5′-GCTGAGCGACAGTTCCTTCT-3′ |
| Mouse SOX2-F | 5′-CAGCATGTCCTACTCGCAGC-3′ |
| Mouse SOX2-R | 5′-GGAGTGGGAGGAAGAGGTAAC-3′ |
| Mouse OCT4-F | 5′-CGGAAGAGAAAGCGAACTAGC-3′ |
| Mouse OCT4-R | 5′-ATTGGCGATGTGAGTGATCTG-3′ |
| Mouse β-actin-F | 5′-GATTACTGCTCTGGCTCCTAG-3′ |
| Mouse β-actin-R | 5′-GACTCATCGTACTCCTGCTTG-3′ |
| Mouse Gammaproteobacterial-F | 5’-CGTYGGAATCTGGCCTCTAGA-3’ |
| Mouse Gammaproteobacterial-R | 5’-AGATTAGCTCCGCATCGCTG-3’ |
| Mouse Enterobacteriaceae-F | 5’-ACCTGGGTACWACCAACTCTTGTGT-3’ |
| Mouse Enterobacteriaceae-R | 5’-GTCACTGCCTGACGTTTAGC-3’ |
| Mouse Fusobacteriales-F | 5’-CGGATCGTAAAGCTCTGTTGTAAG-3’ |
| Mouse Fusobacteriales-R | 5’-GCCGTCCCTTTCTGGTAAG-3’ |

Note: F, forward; R, reverse; FOXQ1, Forkhead box Q1; SIRT1, sirtuin 1
