# Supplementary material for: FOXQ1-mediated SIRT1 upregulation enhances stemness and radio-resistance of colorectal cancer cells and restores intestinal microbiota function by promoting β-catenin nuclear translocation
Source: J Exp Clin Cancer Res. 2022 Feb 19;41:70. doi: 10.1186/s13046-021-02239-4 (PMC8857837; doi:10.1186/s13046-021-02239-4)
Supplement: Supplementary file 3 — Additional file 3: Supplementary Table 2. Correlation between FOXQ1 expression and clinicopathological features of CRC patients. Note: The data were nominal data, which was analyzed by chi-square test. The sample size (n) was 83, and p < 0.05 means that the difference was of statistical significance. [file 13046_2021_2239_MOESM3_ESM.docx]

**Supplementary Table 2 Correlation between FOXQ1 expression and clinicopathological features of CRC patients**

| Clinicopathological features | Cases (n=83) | Expression of FOXQ1 | | *P value* |
| --- | --- | --- | --- | --- |
|  |  | High (n=41) | Low (n=42) |  |
| Age (years) |  |  |  |  |
| ≤ 60 | 50 | 23 | 27 | 0.505 |
| > 60 | 33 | 18 | 15 |  |
| Gender |  |  |  |  |
| Male | 47 | 27 | 20 | 0.122 |
| Female | 36 | 14 | 22 |  |
| Tumor size |  |  |  |  |
| ≤ 5 cm | 57 | 23 | 34 | 0.019 |
| > 5 cm | 26 | 18 | 8 |  |
| TNM staging |  |  |  |  |
| Ι ~ Ⅱ | 51 | 20 | 31 | 0.025 |
| Ⅲ | 32 | 21 | 11 |  |
| Lymphatic metastasis |  |  |  |  |
| Y | 32 | 21 | 11 | 0.025 |
| N | 51 | 20 | 31 |  |

Note: The data were nominal data, which was analyzed by chi-square test. The sample size (n) was 83, and *p* < 0.05 means that the difference was of statistical significance.
